# A novel approach to peritonitis

**DOI:** 10.4102/jcmsa.v4i1.334

**Published:** 2026-08-29

**Authors:** Tshepang A. Motsepe

**Affiliations:** 1 Department of Surgery, School of Clinical Medicine, Faculty of Health Sciences, University of the Witwatersrand, Johannesburg, South Africa

**Keywords:** disease classification, HIV infections, peritonitis, South Africa, tuberculosis

## Abstract

Peritonitis is a deadly abdominal condition seen across many surgical disciplines, with causes including appendicitis, perforated peptic ulcers, and abdominal trauma. In the South African context, peritonitis is largely influenced by tuberculosis (TB), human immunodeficiency virus (HIV), and widespread malnutrition linked to socioeconomic challenges. Although peritonitis is traditionally classified into primary, secondary, and tertiary types, the addition of quaternary peritonitis could reflect the severe complication risks associated with recalcitrant, systemic, or locally advanced disease. This enhanced grading system, tailored to the local setting, may assist clinicians to accurately assess the severity of peritonitis, improve risk stratification, optimise resource allocation, and ultimately lead to better patient outcomes.

## Introduction

Peritonitis is a critical abdominal complication seen across multiple surgical disciplines due to its association with intra-abdominal and pelvic organs.^1,2^ It commonly arises from appendicitis, perforated peptic ulcers, uro-gynaecological conditions, and abdominal trauma.^1,2^ Aetiologies include bacterial or fungal infections and may occur spontaneously in patients with liver or kidney failure (e.g. spontaneous bacterial peritonitis).^1,2^ Local complications include intra-abdominal collections, enterocutaneous fistulae, and surgical site infections. Systemically, it may precipitate septic shock and cardiopulmonary arrest, acute respiratory distress syndrome, multiple organ failure, and ultimately death.^1,2^

Peritonitis holds clinical relevance in South Africa, particularly amid tuberculosis (TB), human immunodeficiency virus (HIV), and endemic malnutrition influenced by socioeconomic factors.^3,4,5,6^ Abdominal TB, a broader term encompassing infection of the terminal ileum, ileocaecal region and peritoneum, may manifest as terminal ileitis, peritonitis, or enteritis.^5^ The more frequent presentation of TB terminal ileitis is characterised by fibrosis and occlusion at the terminal ileum.^5^ Patients typically exhibit overt small bowel obstruction, often accompanied by constitutional symptoms.^5^ This condition may progress to perforation, resulting in secondary peritonitis and a high risk of mortality.^5^ Surgical intervention relieves the obstruction, complemented by antituberculosis therapy. Tuberculous (TB) peritonitis in its pure form is an extrapulmonary TB and primary peritonitis, in which *Mycobacterium tuberculosis* infects the peritoneal lining in isolation.^5^ It typically arises from haematogenous dissemination or reactivation of latent TB foci within the peritoneum, often seeded from previous pulmonary infection.^5^ The mainstay of treatment of TB peritonitis is antituberculosis medications, without surgery.^5^ However, diagnosing abdominal TB remains challenging due to its insidious onset, variable presentations, and the overlap with other abdominal conditions.^5^

Infection with HIV and malnutrition significantly elevate the risk of anastomotic breakdown following surgery, resulting in secondary peritonitis.^3,4,5,7^ A low peri-operative CD4 count (< 250 cells/μL) is a strong predictor of anastomotic leaks, reflecting a compromised healing ability and increased risk of complications.^7^ Malnutrition, often indicated by low serum albumin (< 30 g/L) as a surrogate marker, significantly increases the likelihood of anastomotic leaks, septic collections, and surgical site infections.^6^ Serum albumin levels may fluctuate due to confounding conditions like infections, burns, soft tissue injuries, renal disease, or liver dysfunction, reducing its reliability as a standalone malnutrition indicator.^6^

The demographic and clinical landscape of peritonitis in South Africa is greatly shaped by HIV, TB and poverty, which together contribute to poor health outcomes.^1,3,5,6^ Given the high prevalence of abdominal sepsis and its associated mortality,^3^ alongside the limitations of existing classification systems in fully characterising peritonitis, a robust clinical framework is essential, one that unambiguously quantifies peritonitis severity and promotes early, decisive surgical intervention. This need underpins the development of the Johannesburg classification (JC) system.

## Peritonitis grading systems

Peritonitis grading systems are essential tools for risk stratification, allowing clinicians to categorise patients according to disease severity and predict morbidity and mortality. These systems assist in determining the appropriate level of care, including the need for intensive care unit (ICU) admission in patients at high risk of complications such as organ failure, wound sepsis, pulmonary infection, and death.^8^ Furthermore, they provide a standardised framework for comparing patient cohorts, evaluating therapeutic interventions, and designing clinical trials (see Table 1).

**TABLE 1:** Comparison of the grading systems of peritonitis.

Classification domains	Johannesburg	Mannheim	Björck	Hamburg
Duration of peritonitis	Clearly mentioned, with defined cut-off values of ˂2 days for secondary peritonitis, 2–7 days for tertiary peritonitis, and > 7 days for quaternary peritonitis.	Included as a risk factor (duration ≥ 24 h).	Not captured in the classification system.	Poorly defined; however, tertiary peritonitis is described as occurring after 48 h post attempts at addressing the source of sepsis.
Local complications	Mentioned with enteric leaks beyond 7 days and enterocutaneous fistulae being associated with quaternary peritonitis.	Not clearly indicated. However, generalised peritonitis mentioned as weighing 6 points.	Included, uniquely appreciating the role of visceral fixation to abdominal wall in its grading. Contamination, enteric leaks and enteroatmospheric fistulae are recognised as local abdominal complication.	Secondary peritonitis subclassified with various aetiologies under A: perforation peritonitis, B: post-operative peritonitis, C: post-traumatic peritonitis.
Systemic features of sepsis	Clinical parameters of sepsis mentioned, each with defined cut-off values.	Mentioned with organ failure (e.g. shock, renal failure, respiratory insufficiency) weighing 7 points.	Not captured in the classification system.	Not captured in the classification system.
Demographic and/or clinical parameters	Demographic parameters not included; only clinical indicators of systemic sepsis are included.	Age and sex mentioned, with age > 50 years and female sex both weighting 5 points.	Not captured in the classification system.	Not captured in the classification system.

The Mannheim Peritonitis Index (MPI) is one of the most widely used scoring systems for assessing the severity of secondary peritonitis and predicting mortality. It incorporates eight risk factors, including demographic, physiological, and disease-related variables, with a maximum score of 47. Scores below 21 are associated with minimal mortality risk, scores between 21 and 27 indicate moderate risk, and scores above 27 are associated with a high likelihood of death. Despite its utility, the MPI primarily focuses on systemic and demographic parameters and does not adequately account for the extent of intra-abdominal disease or local complications.^8^

The Björck classification, in contrast, is used in the context of the open abdomen and categorises patients based on the degree of contamination, the presence of adhesions, and local complications such as fistulae. However, it does not incorporate key clinical variables such as the source of infection, duration of disease, or systemic manifestations of sepsis, limiting its applicability as a comprehensive peritonitis grading system.^9^

The current classification of peritonitis, based on the Hamburg scheme, divides the condition into primary, secondary, and tertiary forms according to aetiology and clinical course.^10^ Although widely utilised, this framework does not clearly account for the duration of disease progression, the severity of systemic sepsis, or the presence of advanced local complications. The JC seeks to address these gaps by incorporating these critical variables and introducing a fourth category to reflect advanced disease (see Table 1).

## Johannesburg classification of peritonitis

Within the JC framework (see Table 2 and Table 3), primary peritonitis refers to infection of the peritoneum arising from an extra-abdominal source, typically via haematogenous or lymphatic spread. It is commonly associated with liver cirrhosis and nephrotic syndrome, where ascites predisposes to infection. The causative organisms are usually monomicrobial, with *Streptococcus pneumoniae* and Group A streptococci being the frequently implicated pathogens. This category also includes TB peritonitis. Management is generally non-operative, relying on targeted antimicrobial or antituberculosis therapy, where appropriate.^1,2,10^

**TABLE 2:** Abbreviated Johannesburg classification of peritonitis.

JC categories of peritonitis	Short description
Primary peritonitis	Extra-abdominal origin
Secondary peritonitis	Intra-abdominal origin, ˂ 2 days
Tertiary peritonitis	Intra-abdominal origin, 2–7 days
Quaternary peritonitis	Systemic sepsis, or local abdominal complications, or > 7 days

JC, Johannesburg classification.

**TABLE 3:** Johannesburg classification of peritonitis.

Primary peritonitis (Grade 1)	Peritonitis of extra-abdominal source (i.e. spontaneous bacterial peritonitis).
Secondary peritonitis (Grade 2)	Peritonitis from intra-abdominal aetiology, occurring within 48 h of the inciting event.
Tertiary peritonitis (Grade 3)	Peritonitis from intra-abdominal aetiology, present from 48 h to 7 days. Recurrent peritonitis after adequate abdominal washout during laparotomy, present up to 7 days.
Quaternary peritonitis (Grade 4)	Peritonitis from intra-abdominal aetiology, persisting over 7 days. Peritonitis with systemic features of sepsis:Pyrexia, temperature ≥ 38.5°C or hypothermia, < 36°COngoing tachycardia ≥ 120 bpm, or MAP ˂ 65 mmHg despite fluid challengeAbnormal septic markers,WCC (× 1000/μL) ˂ 4 or ˃ 12,PCT (ng/mL) ≥ 10.0, CRP (mg/dL) ≥ 50New-onset acute kidney injuryAcute neurological deterioration with GCS ≤ 14/15 Peritonitis with ongoing enteric or anastomotic leak, enterocutaneous fistula, or an open abdomen.

Note: Any positive description of peritonitis will be assigned the appropriate grade based on the Table 3.

MAP, mean arterial pressure; WCC, white cell count; PCT, procalcitonin; CRP, C-reactive protein; GCS, Glasgow Coma Scale score.

Secondary peritonitis, the most common form encountered in clinical practice, results from direct contamination of the peritoneal cavity due to perforation or rupture of a hollow viscus. Common causes include perforated appendicitis, peptic ulcer disease, colonic perforation, and ischaemic bowel. Post-operative peritonitis, mostly due to anastomotic leaks, also falls within this category. According to the JC, secondary peritonitis develops within 48 h of the inciting event and typically necessitates surgical source control.^1,2,10^

Tertiary peritonitis remains a poorly defined clinical entity within the current classification system. This category encompasses a broad spectrum of disease severity, ranging from relatively mild presentations to pre-arrest clinical state. The JC simplifies this state of recalcitrant peritonitis into tertiary and quaternary forms. It defines it as a persistent or recurrent intra-abdominal infection that either continues between 2 days and 7 days or recurs within 7 days following initial surgical intervention and sepsis control measures. The JC is not a replacement of the current framework; rather, it is an enhancement. The distinction between tertiary and quaternary peritonitis in the JC is duration, from the above context. Although tertiary peritonitis has traditionally been considered to persist beyond 48 h, the JC further refines this definition by restricting it to within 7 days, with quaternary peritonitis defined as persistence beyond 7 days.

Quaternary peritonitis also refers to the presence of intra-abdominal infection accompanied by systemic sepsis, characterised by pyrexia, tachycardia, elevated inflammatory markers, acute kidney injury, and impaired level of consciousness. Therefore, the duration of infection is not regarded in this context.

Peritonitis associated with an enteric leak that is surgically corrected within 48 h is classified as secondary peritonitis. Tertiary peritonitis refers to cases in which the enteric leak is surgically managed after 48 h but within 7 days. Quaternary peritonitis describes intra-abdominal infection with ongoing unaddressed enteric leak beyond 7 days, a continuously draining enterocutaneous fistula, or bowel conditions that preclude definitive abdominal closure.

Management of quaternary peritonitis typically requires ICU care, ventilatory support, intravenous fluids, vasoactive agents, appropriate antimicrobials, prompt source control, and early enteral nutrition. This entity represents the terminal phase of the disease and carries high mortality.^1,2,10^

The JC further defines recalcitrant peritonitis as intra-abdominal infection persisting beyond 48 h, encompassing both tertiary and quaternary categories. This condition is frequently driven by resistant or opportunistic pathogens, including *Candida* species, *Enterococcus*, and coagulase-negative staphylococci, and may be exacerbated by missed injuries or ongoing contamination from unrecognised micro-perforation of a hollow viscus.

## Discussion

The key principle underpinning the JC is the recognition that the duration of disease prior to effective source control significantly influences outcomes. Early intervention, particularly within the first 24 h, is associated with improved survival and reduced complication rates. Delays beyond this period are linked to a substantial increase in morbidity and mortality. Mortality rates rise significantly after 48 h and may range between 14% and 50%, depending on the clinical context. Evidence suggests that outcomes deteriorate exponentially as the duration of untreated peritonitis increases.^11^

The progression of peritonitis to systemic sepsis represents a critical turning point in the disease course. While mortality in otherwise healthy individuals with early, appropriately managed peritonitis may be as low as 6% – 10%, this increases markedly to 35% – 40% following the onset of septic shock and organ dysfunction. The development of renal failure, respiratory compromise, or neurological impairment further worsens prognosis.^12^

Peritonitis accompanied by systemic sepsis reflects a disseminated pathological process extending beyond the abdominal cavity, with involvement of multiple organ systems.^12^ The JC framework identifies key indicators of multiple organ dysfunction secondary to sepsis, including elevated sepsis-related biomarkers, haemodynamic instability characterised by significant tachycardia and reduced mean arterial pressure (MAP), the development of acute kidney injury, and acute neurological deterioration. However, additional organ system involvement such as acute hepatic failure and acute respiratory failure may also be involved.^12^ These are not formally incorporated into the JC framework to maintain its simplicity and clinical utility. Furthermore, as multiorgan failure typically occurs concurrently across several organ systems, abnormalities in these additional parameters often coincide with those already captured within the framework.

Local complications also play a significant role in determining outcomes. Intra-abdominal abscesses, enteric fistulae, and open abdominal states are associated with high morbidity and mortality. Post-operative peritonitis carries particularly poor outcomes, with mortality rates reported as high as 50% – 60%. These complications create a hostile intra-abdominal environment, complicating surgical management and perpetuating the cycle of local infection and systemic inflammation.^9,13^

The JC acknowledges these factors and aims to provide a more comprehensive and clinically relevant framework for assessing peritonitis severity. While not intended to replace clinical judgement, it offers a structured approach to disease stratification that may improve decision-making and patient outcomes. Importantly, it remains a conceptual model that requires validation through large-scale prospective studies.

The JC framework is relevant for peritonitis, its quantification, and management according to clinical context. There are numerous grading systems in the literature, each designed to achieve a specific mandate. No single framework can realistically encompass all possible variables and confounders without becoming overly complex, broad, and unfocused. The SOFA (Sequential Organ Failure Assessment) and APACHE (Acute Physiology and Chronic Health Evaluation) primarily measure acute organ dysfunctions and outcomes in critically ill patients;^14^ in contrast, the JC, Hamburg, MPI, and Björck classifications focus more specifically on the abdominal pathology.

## Conclusion

South Africa carries a significant burden of peritonitis as a complication of abdominal surgery. This burden is compounded by socioeconomic factors, including malnutrition, and by the high prevalence of HIV and TB, both of which negatively influence post-operative recovery. Existing classification systems do not adequately capture the full spectrum of peritonitis severity, underscoring the need for a robust clinical framework that can clearly stratify disease severity and facilitate timely, decisive surgical management; hence the development of the JC system.
